# (R)evolution of the Standard Addition Procedure for Immunoassays

**DOI:** 10.3390/bios13090849

**Published:** 2023-08-25

**Authors:** Monika Conrad, Peter Fechner, Günther Proll, Günter Gauglitz

**Affiliations:** Institute of Physical and Theoretical Chemistry (IPTC), Eberhard Karls Universität Tübingen, Auf der Morgenstelle 18, 72076 Tübingen, Germanyguenther.proll@uni-tuebingen.de (G.P.); guenter.gauglitz@uni-tuebingen.de (G.G.)

**Keywords:** (bio-)sensor, calibration-free, immunoassay, label-free, reflectometric interference spectroscopy (RIfS), standard addition

## Abstract

A new method to transfer the standard addition procedure for concentration determination to immunoassays with non-linear calibration curves was developed. The new method was successfully applied to simulated data and benchmarked against a state-of-the-art algorithm, showing a significantly improved performance with improvement factors between 2 and 192. The logit function was used to transform the immunoassay signal response of test samples spiked with known analyte concentrations. The relationship between logit(signal) and log-transformed estimated total analyte concentration is linear if the estimated total analyte concentration is correct. Finally, the new method was validated experimentally using different assays in varying, relevant complex matrices, such as serum, saliva, and milk. Different concentrations of testosterone and amitriptyline between 0.05 and 3.0 µg L^−1^ were quantified using a binding inhibition assay in combination with reflectometric interference spectroscopy (RIfS) as the transduction principle. The sample concentration was calculated using a numerical method. Samples could be quantified with recoveries between 70 and 118%. The standard addition method accounts for individual matrix interference on the immunoassay by spiking the test sample itself. Although the experiments were carried out using RIfS, the method can be applied to any immunoassay that meets the analytical requirements.

## 1. Introduction

Nowadays, it is hard to imagine areas such as food [1,2], environmental analysis [3,4,5], or diagnostics [6,7] without biosensors [8]. Traditionally, these biosensors rely on antibodies [9] or enzymes [10,11] as recognition elements but can also make use of more exotic proteins [12] or aptamers [13,14]. In particular, biosensors are characterized by the fact that they are easy to use and usually inexpensive while at the same time offering high specificity and sensitivity in addition to low limits of detection, which makes them the ideal platform for detecting a wide range of analytes. However, the influence of matrix components on the analytical result must always be considered and often negatively influences the analytical performance of an assay. For quantitative analysis, a calibration curve is usually measured beforehand, which includes a zero calibrator and known analyte concentrations in solution [15]. The solution for compensating matrix effects during the calibration measurements is to mimic the complexity of the matrix because components in the sample can lead to non-specific effects [16]. As diet, supplements, medications, and wash products can cause matrix effects in immunoassays [17], it is difficult to account for them while measuring the calibration curve. Additionally, adapting and calibrating an immunoassay to any relevant matrix is time-consuming and expensive in the best case and impossible for some cases, such as personalized medicine. To solve these problems in classical analytics, the standard addition method is often applied [18].

The standard addition method was first used in polarography to determine zinc by Hohn (1937) [19]. He employed a two-level design, measuring first the sample and second the sample with added standards. About 20 years later, the standard addition method was applied to spectroscopy [20]. In spectroscopy, successive dilutions were prepared and measured. In 1954, the standard addition method was also used in X-ray fluorescence analysis to determine niobium and tantalum in ores [21]. Around the same time in 1955, the standard addition method was first applied to determine an analyte (strontium) in a complex natural aqueous medium (seawater) [22]. Details on the origin of the standard addition method can be found in Kelly et al. (2011) [23].

The conventional standard addition method is only applicable to systems with linear responses. It is used to determine the concentration of the analyte in its matrix. A sample is divided into aliquots of equal volume. The aliquots are spiked with known and varying analyte standards. The concentration is determined as the negative x-intercept [24]. The standard addition method assumes that a zero response is obtained when the total concentration of the analyte is zero [25]. Thus, the conventional standard addition method cannot be used for immunoassays because the obtained calibration curves are neither linear nor cross the origin.

The signal response obtained in immunoassays is—in most cases—sigmoid, given the fact that the concentrations are plotted on a logarithmic scale. The four-parameter logistic fit (1) with the theoretical response at zero concentration $A_{1}$, the theoretical response at infinite concentration $A_{2}$, the inflection point $x_{0}$, and the slope factor p is usually used for fitting calibration curves of immunoassays:

(1) $$y=A_{2}+\frac{A_{1}-A_{2}}{1+{\left(\frac{c}{x_{0}}\right)}^{p}}$$

It is recommended to use five to eight calibration concentrations in duplicate or triplicate [26]. The Hill Equation (2), which is used in pharmacology to describe quantitative drug-receptor relationships, and the four-parameter logistic function are mathematically identical [27] if $A_{2}$ is zero.

(2) $$y=\frac{A_{1}\cdot x_{0}^{\alpha }\;}{c^{\alpha }+x_{0}^{\alpha }}$$

The p value of the four-parameter logistic fit function is equivalent to the Hill coefficient α [28]. The Hill coefficient reflects cooperativity among multiple ligand binding sites [29] and may indicate the number of interacting sites [30].

Pang and Cowen (2017) suggested transferring the standard addition method to sigmoid calibration curves with a novel evaluation method [31]. Known concentrations are added to the sample with unknown concentration. The unknown concentration is estimated, and the sum of known added standard and unknown estimated concentration are logarithmized and plotted as the log of the total estimated concentration on the abscissa. The logarithmized signals are the y-coordinates, and a linear regression is performed. The deviation from linearity is used to optimize the estimated sample concentration. The estimated sample concentration is varied until the best linearity is found, indicating the sample concentration

While the method works in principle, we found that it suffers from some limitations. In our work, we significantly improved the log-log approach of Pang and Cowen (2017) [31] in theory and practice and now propose a novel algorithm for calibration-free concentration determination in immunoassays with the standard addition method. The algorithm is demonstrated with simulated data and applied to real measurements of two analytes in different matrices. The matrices are buffer, saliva, milk, and serum. Measurements were recorded with reflectometric interference spectroscopy (RIfS). The simulated data show that our method significantly improves the log-log approach [31]. The evaluation of real measurements shows that with our logit-log approach, the determination of concentrations in complex matrices works excellently, even if the log-log approach does not lead to any result.

## 2. Materials and Methods

### 2.1. Materials

Common chemicals were purchased from Sigma-Aldrich (Taufkirchen, Germany). The testosterone antibody (monoclonal, clone 14P2C8, host mouse, Invitrogen) was purchased from Thermo Fisher Scientific (Schwerte, Germany). Poly(ethylene glycol) diamine (PEG-DA, MW 2000 Da) and α-methoxy-ω-amino PEG (PEG-MA, MW 2000 Da) were purchased from Rapp Polymere (Tübingen, Germany). Phosphate-buffered saline (PBS, 150 mM sodium chloride, and 10 mM potassium phosphate, pH 7.4) was used as buffer. Sodium dodecyl sulfate (SDS, 0.5%, pH 1.6) was used for regeneration of the sensor surface. Milli-Q water was used in the preparation of all solutions. Milk (1.5% fat, ultra-high temperature pasteurized, Schwarzwaldmilch, Freiburg, Germany) was purchased from a local supermarket. RIfS glass transducers (Schott AG, Mainz, Germany, 1 cm × 1 cm) consisted of a 1 mm glass substrate with a layer of 10 nm Ta_2_O_5_ covered with 330 nm SiO_2_.

### 2.2. Assay—Binding Inhibition Test

The tested analytes amitriptyline and testosterone are small molecules (MW < 1000 Da). Thus, the binding inhibition test is the assay format of choice. As analyte derivatives are covalently bound to the surface, the immunoassay is heterogeneous. RIfS is a label-free technique; therefore, no labeling is required. The sample is preincubated with a defined amount of antibody. After equilibrium conditions are reached, the sample is pumped over the sensor surface. Only antibodies with free paratopes can bind to the immobilized analyte derivatives, resulting in a change in the interference spectrum. The reaction should be mass transport limited for quantitative results. To achieve mass transport limitation, a small amount of antibody and high surface loading should be used. The signal decreases with increasing analyte concentration in the sample.

### 2.3. Simulation

Data for simulations were generated with Matlab R2020b. For signal generation, the sigmoid calibration Function (1) was used. The parameters were $A_{1}=1.0$, $A_{2}=0.0$, $x_{0}=10^{-7}M$, p was varied. The values for the asymptotes of the calibration function were chosen to be $A_{1}=1.0$, $A_{2}=0.0$ because it is common practice to normalize the obtained signals; thus the signals range between 0 (minimum signal) and 1 (maximum signal). The value of the inflection point is related to the affinity of the antibody used in the immunoassay and was set to $x_{0}=10^{-7}M,$ as this is in the range of typical values for antibodies. The tested concentrations were in the working range of the calibration curves between 10 and 90% inhibition. One hundred logarithmically equidistant sample concentrations in the working range were tested. The added standard concentrations were six concentrations, with the first being zero, then five concentrations logarithmically equidistant in the working range, starting with the concentration that caused 10% inhibition. The signals of the sample with its added standards were generated with the sigmoid calibration curve. The test of linearity was performed with 100 estimated sample concentrations within the working range. The X values for the test were the sum of the estimated concentration and known added standard concentration, which was logarithmized (common logarithm log10). The Y values were the logit (3) or natural logarithm (log) of the signals.

(3) $$logit\left(y\right)=\log\frac{y}{1-y}$$

For the test of linearity, the residual sum of squares (SSres) was calculated for each estimated concentration. The minimum of SSres was determined, which gave the sample concentration.

### 2.4. RIfS Transducer Surface Modification

Transducers for RIfS measurements of testosterone in different matrices were prepared as described in Rau and Gauglitz (2012) [32]. First, the transducers were cleaned with KOH (6 M) for 30 s and activated with piranha (3:2 conc. H_2_SO_4_:H_2_O_2_ (30%)) for 15 min. Then, the transducers were incubated with 3-glycidolxypropyl-trimethoxysilane (GOPTS) for 1 h. For measurements in buffer and milk, PEG-DA (4 g L^−1^) in dichloromethane (DCM) was bound to the GOPTS layer. For measurements in serum and saliva, a polymer mixture of 5% PEG-DA and 95% PEG-MA was used. Mixing PEG-DA with PEG-MA reduces the surface capacity as only PEG-DA can form a covalent bond to the antigen derivative. Optimizing the surface properties is an important step to improve assay performance. The PEG solution (20 µL) was pipetted onto each slide, and the transducers were incubated overnight at 70 °C. Subsequently, the transducers were rinsed with H_2_O and dried under nitrogen. A solution of testosterone-3-(O-carboxymethyl)oxime (100 g L^−1^) and N,N’-diisopropyl-carbodiimide (DIC) (0.15 L L^−1^) in dimethylformamide (DMF) was prepared and 10 µL pipetted onto half of the slides. The slides were incubated as sandwiches overnight in a DMF vapor-saturated chamber. Then, the transducers were washed with DMF and H_2_O and dried under nitrogen. The transducers for the measurements of amitriptyline were prepared as described in Conrad et al. (2021) [33]. After the modification with PEG-DA, glutaric acid (10 µL 670 g L^−1^) in DMF was pipetted onto half of the transducers. The transducers were covered with another transducer in a DMF vapor-saturated chamber. After incubation for 6 h, the transducers were rinsed with DMF and H_2_O, and dried under nitrogen. For carbodiimide-mediated coupling, the transducers were covered in a solution of DIC (302 mL L^−1^) and N-hydroxysuccinimide (NHS) (150 g L^−1^) in a DMF vapor-saturated chamber for 4 h. The transducers were cleaned with DMF and acetone and dried under nitrogen. For the last step, the transducers were incubated with nortriptyline (2 g L^−1^) in H_2_O in an H_2_O vapor-saturated chamber overnight. Finally, the transducers were washed with H_2_O and dried under nitrogen.

### 2.5. Reflectometric Interference Spectroscopy

RIfS is a label-free and time-resolved biosensor method that is based on direct optical detection [34]. A polymer fiber guides the white light from a halogen lamp to the flow cell with the transducer. At phase boundaries, part of the light is transmitted, and part is reflected. Superimposition results in an interference spectrum. A change in optical thickness causes a shift in the interference spectrum. The optical thickness is the product of physical thickness and refractive index. More details about the method and the setup can be found in the literature [35].

### 2.6. RIfS Measurements

For a binding inhibition test, the sample was incubated with the antibody at RT, 30 min for amitriptyline and 10 min for testosterone. Only antibodies with free binding sites can bind to the antigen on the surface. To obtain quantitative results, the binding of the antibody to the surface should be mass transport-limited. Measurements were performed as described in Table 1.

**Figure 1:**
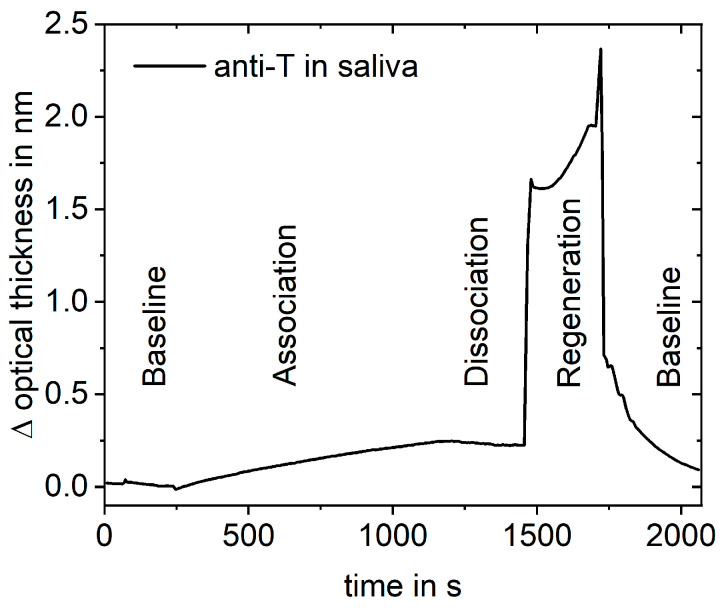
Example measurement showing a typical binding curve: baseline with buffer, association with preincubated sample, dissociation with buffer, removal of antibodies with regeneration solution, baseline with buffer.

For measurements of testosterone in buffer, 400 µL sample with 3 µg L^−1^ testosterone in PBS were mixed with 50 µL standard solution and 50 µL anti-testosterone (25 mgL^−1^). For measurements of testosterone in milk (1.5% fat), a 900 µL sample with 0.3 µg L^−1^ testosterone in milk was mixed with 80 µL standard solution and 20 µL anti-testosterone (25 mg L^−1^). For measurements of testosterone in saliva (artificial saliva for pharmaceutical research), a 630 µL sample with 0.05 µg L^−1^ testosterone in saliva was mixed with 7 µL ovalbumin (OVA) (10 g L^−1^), 11.2 µL anti-testosterone (25 mg L^−1^), standard solution, and PBS (10×, 1.5 M NaCl, 100 mM KH_2_PO_4_, pH 6.8) to a final volume of 700 µL. For measurements of testosterone in fetal calf serum (FCS), a 70 µL sample with 10 or 1.0 µg L^−1^ testosterone in FCS was mixed with 7 µL OVA (10 g L^−1^), 5.6 µL anti-testosterone (25 mg L^−1^), standard solution, and PBS to a final volume of 700 µL. For measurements of amitriptyline in FCS, a 70 µL sample with 2.5 µg L^−1^ amitriptyline in FCS was mixed with 7 µL OVA (10 g L^−1^), 1.166 µL anti-amitriptyline (1.2 g L^−1^), standard solution, and PBS to a final volume of 700 µL. An example measurement is shown in Figure 1.

For measurements in buffer, saliva, and serum, the binding signal during the association phase is linear, and its slope was evaluated. Because of Tyndall scattering, this was not possible for milk; instead, the optical thickness at the end of the dissociation phase was evaluated. In each matrix, a blank measurement without antibodies was performed. The blank measurement was deducted from the others. To obtain the maximum binding signal, an antibody solution without analyte was measured and used for the normalization.

## 3. Results

### 3.1. Simulations

To test the algorithm with flawless data, signals were simulated. The chosen p values for the simulations with the four-parameter logistic fit function (1) were 0.15, 1.0, and 3.2 because the lowest observed Hill coefficient was 0.15 [30], the highest value was 3.2 [27], and the typical value was around 1. The other parameters were kept constant.

**Figure 2:**
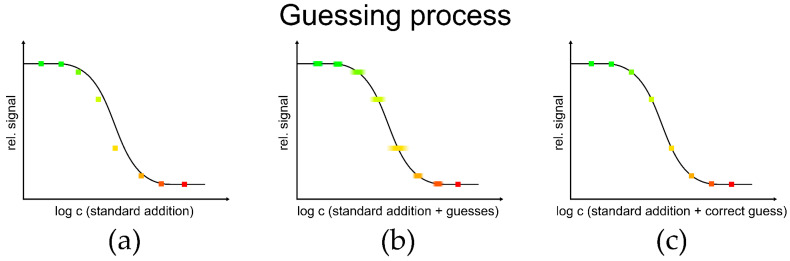
Guessing process: (**a**) relative signal vs. logarithmized added standard concentration, (**b**) relative signal vs. logarithmized added standard concentration + guesses. The blurred points illustrate the different guesses of the concentration. (**c**) Relative signal vs. logarithmized added standard concentration + correct guess. The different colours of the points illustrate different added standard concentrations.

**Figure 3:**
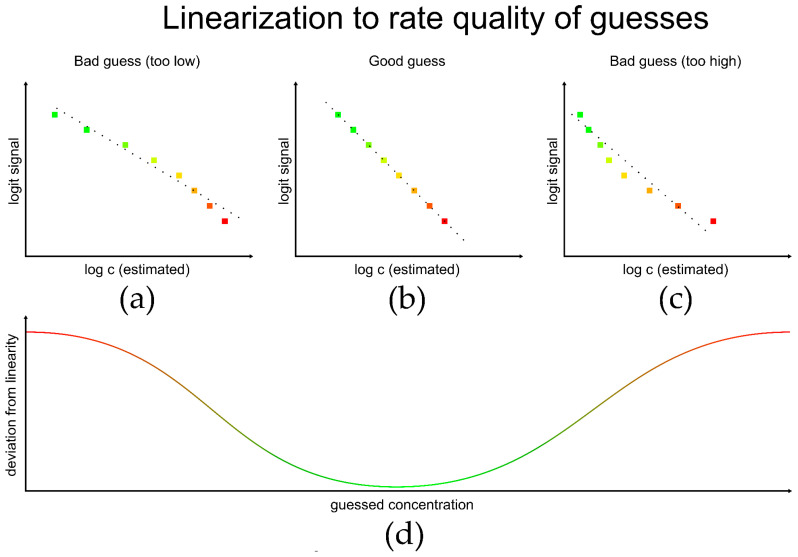
Linearization to rate quality of guesses: top: logit signal vs. logarithmized estimated sample concentration; the differently colored squares correspond to the differently concentrated added standards; the dotted line describes the linear fit; (**a**) guess is too low, the plot is not linear; (**b**) good guess: the plot is linear, (**c**) guess is too high, the plot is not linear; (**d**) plot of deviation from linearity vs. guessed concentration, at the minimal deviation (green) from linearity the correct sample concentration is found.

The simulated signals were evaluated with the log-log approach [31] and the logit-log approach. The established algorithm is first described for creating and evaluating simulated data in Figure 2 and Figure 3. It involves a guessing process to determine the concentration of the sample. A sample with an unknown sample concentration is spiked with different standard concentrations. For a binding inhibition test, decreasing signals are obtained with increasing concentration. When plotting the relative signals against the logarithmized standard concentration, the signals deviate from the sigmoid curve (Figure 2a). In the guessing process for determination of the sample concentration, an estimated sample concentration is added to the known added standard concentration. When plotting the relative signal against the logarithmized estimated total sample concentration (standard addition + guesses) (Figure 2b), the signals will follow the sigmoid curve at the correct guess (Figure 2c).

The developed algorithm uses linearization to rate the quality of guesses. When plotting logit(signal) against the logarithmized concentration, the best linearity will be obtained at the correctly estimated concentration (Figure 3b). At a total estimated concentration that is too low or too high, deviations from linearity will be observed (Figure 3a,c). To assess linearity, a linear regression is performed, and an indicator for the deviation from linearity, e.g., SSres, is determined for the different guessed concentrations. The sample concentration is found at the minimum deviation of linearity.

The algorithm was applied to signals simulated with the four-parameter logistic function for three different p values. The results for simulated data (Table 2) show that the logit-log approach works in the entire tested concentration range for all concentrations, while the log-log approach can only determine concentrations in a small part of the calibration curve. The part of the tested concentrations where the standard addition log-log approach calculates concentrations correctly lies at high concentrations, where the signal is strongly inhibited. For its application in immunoassays, it is a disadvantage if only a fraction of the dynamic range of an assay can be evaluated. Thus, the standard addition logit-log approach is better suited for determining concentrations in immunoassays in general.

To show the general applicability of the evaluation method independent from the chosen assay format, two approaches were tested with simulated signals, which can be obtained in a direct or sandwich assay. The dose-response curve for these test formats can be described with the same function as the one for a binding inhibition test or competitive format, but the signal increases with increasing analyte concentration. The result was the same as for the simulations shown above. The standard addition log-log approach fails at calculating the concentrations, while the standard addition logit-log approach can be applied across the entire tested range.

### 3.2. Measurements

To prove the performance of this novel algorithm, it was applied to real data. Testosterone was measured in various complex matrices as an example analyte. The measurements were conducted with RIfS, a well-established, label-free method. Testosterone was measured in PBS, saliva, fetal calf serum, and milk. Different sample concentrations were prepared, and the added standard concentrations were in the dynamic range of the assay. To ensure that the method is easily transferable to other analytes, the analyte amitriptyline was chosen as an additional analyte, and its concentration was measured in serum as a complex matrix. As the analyzed analytes are small molecules, the binding inhibition test was used. The sample was incubated with antibody solution; then the solution was pumped over the sensor surface, where the antigen was immobilized. The number of free antibody paratopes depends on the analyte concentration. The higher the analyte concentration, the more paratopes are inhibited and can no longer bind to the sensor surface. Thus, the observed signal decreases with increasing analyte concentration.

**Figure 4:**
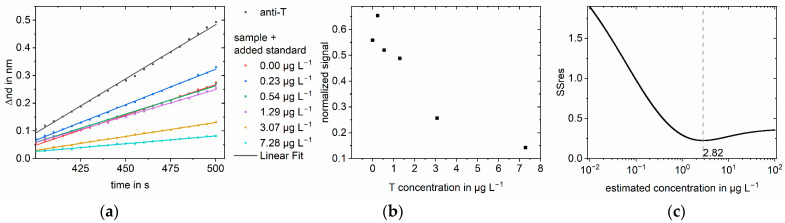
Standard addition for 3.0 µg L^−1^ testosterone in PBS: (**a**) Linear regression of part of the association phase for anti-testosterone (anti-T) blank measurement and the sample with different added standards. The change in optical thickness is plotted against time. The measured value was recorded every five seconds; (**b**) Normalized slopes at different added standard concentrations. The slopes were normalized to the blank antibody measurement; (**c**) SSres at different estimated sample concentrations calculated with logit-log with minimum indicating the sample concentration.

As an example, the applied method is explained in detail for testosterone in buffer; however, the same principle can be applied to any immunoassay. A sample concentration of 3 µg L^−1^ testosterone was chosen. Six different standard concentrations from 0.00 to 7.28 µg L^−1^ were added to the sample. After mixing with anti-testosterone and preincubation, the samples were measured in RIfS. In addition to the sample solutions, anti-testosterone was measured without testosterone to obtain the maximum signal for normalization. A measurement consisted of baseline, association, dissociation, regeneration, and baseline. In Figure 4a, the evaluated part of the association phase is shown. A linear regression was performed to calculate the respective slopes. The slope decreases with increasing standard concentration. The slopes of the sample measurements were normalized using the anti-testosterone measurement. Figure 4b shows the normalized slopes plotted against the concentration of the added standard. For an added standard concentration of 0.00 µg L^−1^, the sample was measured without adding testosterone standard solution, and the obtained normalized signal was inhibited by 44% compared to the measurement of anti-testosterone. The inhibition increases to 86% for the highest added standard concentration of 7.28 µg L^−1^. The normalized slopes were used as signal values in the algorithm to calculate the Y values of the linear regression, logit(normalized signal) for our approach, and log(normalized signal) for the log-log approach. The X values for the linear regression were calculated as the logarithmized sum of the known added standard concentration and an estimated sample concentration. The estimated sample concentrations were 1000 logarithmically equidistant concentrations between 0.01 and 100 µg L^−1^. The number of tested concentrations is arbitrary, but one has to ensure that the tested concentrations are close enough in the region of the sample concentration. For each estimated sample concentration, a linear regression was performed, and the SSres was calculated. The sample concentration was found at the minimum of SSres. Figure 4c shows the SSres obtained with the logit-log approach, with the minimum indicating the sample concentration. The minimum for the standard addition logit-log approach was found at 2.82 µg L^−1^, corresponding to a calculated sample concentration of 3.53 µg L^−1^. For the standard addition log-log approach, the found concentration was 6.99 µg L^−1^. Thus, the logit-log approach gives the correct sample concentration of 3.0 µg L^−1^ with an acceptable deviation from the true concentration of 18%, while the log-log approach fails in concentration determination.

**Figure 5:**
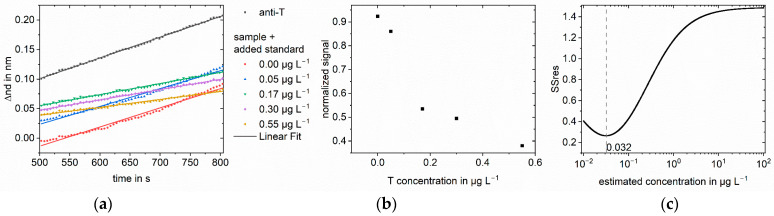
Standard addition for 0.05 µg L^−1^ testosterone in saliva for measurements 9:1 diluted: (**a**) Linear regression of part of the association phase for anti-testosterone (anti-T) blank measurement and the sample with different added standards. The change in optical thickness is plotted against time. The measured value was recorded every five seconds; (**b**) Normalized slopes at different added standard concentrations. The slopes were normalized to the blank antibody measurement; (**c**) SSres at different estimated sample concentrations calculated with logit-log with minimum indicating the sample concentration.

**Figure 6:**
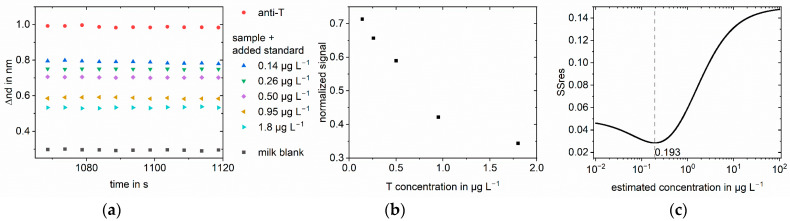
Standard addition for 0.3 µg L^−1^ testosterone in milk for measurements 9:1 diluted: (**a**) Selected part for calculation of the mean change in optical thickness at the end of the dissociation phase for milk blank measurement, anti-testosterone (anti-T) in milk blank measurement and the sample with different added standards. The change in optical thickness is plotted against time. The measured value was recorded every five seconds; (**b**) Normalized signals at different added standard concentrations. The slopes were normalized to the blank antibody measurement; (**c**) SSres at different estimated sample concentrations calculated with logit-log with minimum indicating the sample concentration.

**Figure 7:**
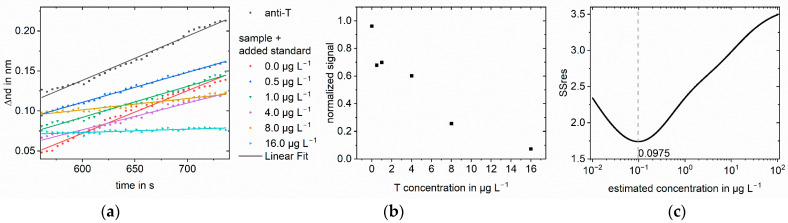
Standard addition for 1.0 µg L^−1^ testosterone in FCS for measurements 1:9 diluted: (**a**) Linear regression of part of the association phase for anti-testosterone (anti-T) blank measurement and the sample with different added standards. The change in optical thickness is plotted against time. The measured value was recorded every five seconds; (**b**) Normalized slopes at different added standard concentrations. The slopes were normalized to the blank antibody measurement; (**c**) SSres at different estimated sample concentrations calculated with logit-log with minimum indicating the sample concentration.

**Figure 8:**
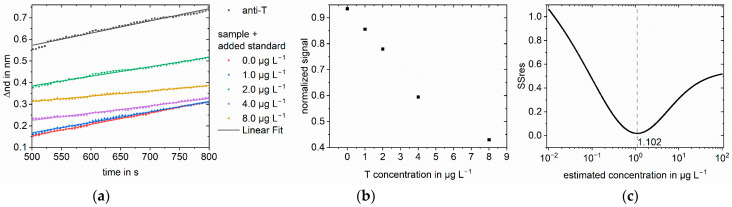
Standard addition for 10 µg L^−1^ testosterone in FCS for measurements 1:9 diluted: (**a**) Linear regression of part of the association phase for anti-testosterone (anti-T) blank measurement and the sample with different added standards. The change in optical thickness is plotted against time. The measured value was recorded every five seconds; (**b**) Normalized slopes at different added standard concentrations. The slopes were normalized to the blank antibody measurement; (**c**) SSres at different estimated sample concentrations calculated with logit-log with minimum indicating the sample concentration.

**Figure 9:**
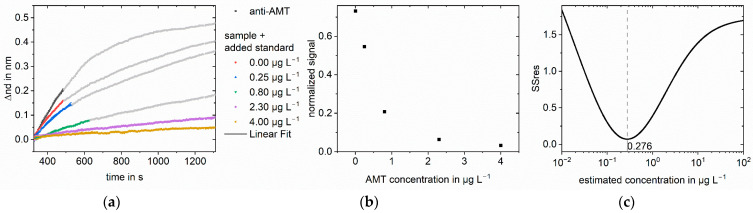
Standard addition for 2.5 µg L^−1^ AMT in FCS for measurements 1:9 diluted: (**a**) Linear regression of mass transport limited part of the association phase for anti-amitriptyline (anti-AMT) blank measurement and the sample with different added standards. The change in optical thickness is plotted against time. The measured value was recorded every five seconds; (**b**) Normalized slopes at different added standard concentrations. The slopes were normalized to the blank antibody measurement; (**c**) SSres at different estimated sample concentrations calculated with logit-log with minimum indicating the sample concentration.

The same method was applied to testosterone in different matrices: saliva (Figure 5), milk (Figure 6), and serum (Figure 7 and Figure 8). For saliva and serum, a blank measurement with the respective matrix as a sample without antibodies was deducted from the other measurements to correct for the change in refractive index. Afterward, the method was the same as for the buffer measurements. For milk, the association phase could not be evaluated because of the strong Tyndall effect in milk. Instead, the optical thickness at the end of the dissociation phase was used. The blank and the anti-testosterone measurement without testosterone were used for the normalization. To show that the method can be transferred to other analytes, amitriptyline in serum was quantified (Figure 9). The method was the same as for the testosterone measurements.

All results are summarized in Table 3. For 3 µg L^−1^ testosterone in buffer, the found concentration for the standard addition log-log approach was 6.99 µg L^−1^, which is equivalent to a recovery of 233%. In contrast, with the standard addition logit-log approach, a concentration of 3.53 µg L^−1^ is determined, which corresponds to a recovery of 118%. This shows that the standard addition logit-log approach gives a concentration that is closer to the true value. An improvement factor of 2 was calculated, demonstrating that, for this case, the standard addition logit-log method is twice as good as the log-log approach. For 10 µg L^−1^ testosterone in serum, no minimum of SSres could be found for the log-log approach. Consequently, no concentration could be determined. The estimated concentrations were in the range of 0.01 to 100 µg L^−1^, containing the sought concentration. The found concentration with the logit-log approach was 11.0 µg L^−1^, which is equivalent to a recovery of 110%. The log-log approach cannot find the correct concentration for any of the measurements. The logit-log approach calculates the sample concentrations in the different matrices with recoveries between 70 and 118%. The superiority of the standard addition logit-log approach over the standard addition log-log approach is illustrated by the improvement factor, which is between 2 and 192.

## 4. Discussion

The simulations show that the quantification with the log-log approach can only be applied to a part of the dynamic range of the calibration curve, while the logit-lot approach can correctly calculate concentrations in the entire working range of the calibration curve. Thus, the log-log approach cannot use the entire dynamic range of the immunoassay. The reason for this is that the logit-log plot is a real linearization of the logistic function. The log-log plot only gives a linear correlation in a small range. It is obvious that the logit-log approach is advantageous to determine concentrations with the standard addition method in practical applications. The results of the simulations show that the standard addition logit-log method can be generally used for quantification in immunoassays with logistic calibration curves. To demonstrate the procedure in real measurements, different analytes were quantified with the standard addition method following the same evaluation procedure.

The RIfS measurements in different matrices were evaluated with the log-log approach and the logit-log approach. In the case of the log-log approach, none of the samples were correctly quantified, while the logit-log approach calculates the sample concentrations in the different matrices with recoveries between 70 and 118% (Table 3), which is a significant improvement in general and analytically a more than acceptable result. In particular, since recovery rates should be between 70 and 130% [35]. The improvement factors of 2 to 192 demonstrate the superiority of the logit-log approach. The results show that the standard addition approach can be transferred to sigmoid calibration curves independent of the sample matrix using a numerical method to test for linearity with the logit-log approach.

Saliva is a complex matrix for biosensors because the components of saliva can interfere with the antibody–antigen binding and bind non-specific to the sensor surface [36]. We successfully quantified 50 pg mL^−1^ testosterone in artificial saliva, which is within the range of usual testosterone concentrations in saliva [37,38,39,40]. Compared to the analysis with a conventional SPR biosensor [41], our method does not require calibration. This can reduce the number of required measurements. Typical testosterone levels in serum for men are between 2.5 and 9.5 µg L^−1^, and for women, between 0.1 and 0.6 µg L^−1^ [42]. Since the tested testosterone concentrations of 1 and 10 µg L^−1^ are within this relevant range, it is proven that the used assay can be applied within the relevant concentration range of real serum samples.

To implement the standard addition approach, the dynamic range of the conventional calibration curve should be known to choose adequate standard concentrations. For adequate dilution of the sample, knowledge of the concentration range of the analyte is necessary. This method can be used for any immunoassay with a sigmoid calibration curve.

## 5. Conclusions

In this work, we present a new and improved algorithm to expand the standard addition method from linear dose-response systems to non-linear behavior. A significant improvement over existing algorithms (log-log approach [31]) is shown in both simulations and—more importantly—real measurements. The accuracy of recovery rates was—at least—improved by a factor of two and, depending on the conditions, by orders of magnitude.

However, there are certain limitations for this new algorithm and where it is superior to calibration: first of all, the assay must be robust enough and have sufficient accuracy. Additionally, methods that are already highly parallelized and automated (microtiter plate-based enzyme-linked immunosorbent assay, radioimmunoassay, …) will benefit significantly less due to the possibility of running an internal calibration with minimal loss in performance, e.g., speed.

The strengths of this algorithm become obvious when dealing with different complex matrices, which differ too much to be calibrated individually, and methodologies, which are limited in throughput. Therefore, a multiplexed point-of-care testing (POCT) assay such as the lateral flow assay, could significantly benefit from this algorithm leaping forward from being semi-quantitative (at most) to be a real competitor in quantitative POCT analytics.

## Figures and Tables

**Table 1:** RIfS program for different matrices with volume V and velocity v.

Matrix	Buffer		Milk		Saliva		Serum	
	V in µL	v in µL s^−1^	V in µL	v in µL s^−1^	V in µL	v in µL s^−1^	V in µL	v in µL s^−1^
Baseline	100	0.4	240	2	60	0.5	100	0.5
Association	100	0.4	800	2	450	0.5	450	0.5
Dissociation	100	1	6000	20	360	2	1200	2
			120	2				
Regeneration	200	5	800	2	400	2	400	2
Baseline	150	0.4	300	2	100	0.5	100	0.5

**Table 2:** Results for simulation of the standard addition method using the log-log or logit-log approach. Percentage of correctly determined concentrations of tested concentrations and concentration range where the respective method works are given.

Method	Log-log		Logit-log	
p	% Correct	M Correct Range	% Correct	M Correct Range
0.15	24	2.0 × 10^−4^–0.23	100	4.3 × 10^−14^–0.23
1.0	3	7.9 × 10^−7^–9.0 × 10^−7^	100	1.1 × 10^−8^–9.0 × 10^−7^
3.2	7	1.8 × 10^−7^–2.0 × 10^−7^	100	5.0 × 10^−8^–2.0 × 10^−7^

**Table 3:** Found concentrations and recoveries for testosterone at different concentrations in different matrices and amitriptyline in serum. The found concentration was determined by using the standard addition log-log or standard addition logit-log approach for a test of linearity at different estimated concentrations. The found concentration was the estimated concentration with best linearity determined as the minimum of SSres. The improvement factor was calculated as (log-log recovery)/(logit-log recovery).

Sample Concentration in µg L^−1^	Analyte	Matrix	Found Concen-tration in µg L^−1^ Log-log	Recovery in % Log-log	Found Concen-tration in µg L^−1^ Logit-log	Recovery in % Logit-log	Improvement Factor
3.0	Testosterone	Buffer	6.99	233	3.53	118	2
0.3	Testosterone	Milk	0.607	202	0.214	71	3
0.05	Testosterone	Saliva	0.124	249	0.035	70	4
1.0	Testosterone	Serum	187	18740	0.975	110	192
10	Testosterone	Serum	-	-	11.0	98	∞
2.5	Amitriptyline	Serum	7.22	289	2.76	111	3

-: No minimum of SSres was found.

## Data Availability

The data presented in this study are contained within this article.
